# Does the Relation between Rapid Automatized Naming and Reading Depend on Age or on Reading Level? A Behavioral and ERP Study

**DOI:** 10.3389/fnhum.2018.00073

**Published:** 2018-02-22

**Authors:** Marjolaine Cohen, G. Mahé, Marina Laganaro, Pascal Zesiger

**Affiliations:** ^1^Department of Psychology, Université de Genève, Geneva, Switzerland; ^2^Department of Psychology, SCALab (UMR CNRS 9193), University of Lille, Lille, France

**Keywords:** reading, rapid automatized naming (RAN), ERP, children, French

## Abstract

Reading predictors evolve through age: phonological awareness is the best predictor of reading abilities at the beginning of reading acquisition while Rapid Automatized Naming (RAN) becomes the best reading predictor in more experienced readers (around 9–10 years old). Those developmental changes in the relationship between RAN and reading have so far been explained in term of participants' age. However, it should be noted that in the previous experiments age always co-vary with participants reading level. It is thus not clear whether RAN-reading relationship is developmental in nature or related to the reading system itself. This study investigates whether the behavioral changes in the relationship between RAN and reading and their electrophysiological correlates are related to the chronological age or to the reading level of the participants. Thirty two French-speaking children aged 7–10 years took part to the experiment: they were divided into groups contrasted on age but with similar reading levels and the other way round. Participants performed two reading tasks and four RAN tasks. EEG/ERP was recorded during discrete letter and picture RAN. Behavioral results revealed that alphanumeric RAN is more sensitive to age variations than reading level differences. The inverse profile was revealed for picture RAN, which discriminate poor and good readers among typically developed children within the same age-group. ERPs of both letter and picture RAN differed across age groups whereas only for the picture RAN ERPs differed across reading levels. Taken together, these results suggest that picture RAN is a particularly good indicator of reading level variance independently of age.

## Introduction

Literacy skills are an essential asset in our modern societies as they are critical for academic and professional achievement as well as for social integration. Five years of academic training in a specific orthographic system are necessary to reach an expert reading level (Aghababian and Nazir, 2000), characterized by effortless, rapid, and accurate reading. Despite the special focus placed on reading acquisition over the first school grades, there are huge inter-individual differences in the ease and speed children display in learning to read. Variability in reading skills has been reliably associated with performance in non-reading tasks, and in particular with rapid automatized naming (RAN) tasks. RAN, defined as the ability to name quickly and accurately items displayed on a grid, is a strong predictor of reading skills once children have achieved a certain level of proficiency, usually after the age of 9 years or after Grade 3 (van den Bos et al., 2002; de Jong, 2011). It is however not clear whether the onset of the close RAN-reading relationship is dependent on the degree of expertise in reading, or on the chronological age of the participants, as both variables usually co-vary. The present study aims at investigating whether the RAN-reading relationship is more closely related to the chronological age of the participants, or to their degree of expertise in literacy. An additional insight in the relation between RAN and reading is achieved through the EEG/ERP recording during the RAN tasks, allowing investigating whether the neurophysiological changes due to age and to reading level are the same.

From the 1970s, a wealth of scientific studies has been dedicated to understanding the processes and determinants involved in learning to read, resulting in several consensual statements that we summarize below. First, reading involves both specific written word identification skills, and more general text comprehension skills (Hoover and Gough, 1990). In order to identify written words, the reader is thought to develop two pathways (Coltheart et al., 1993, 2001). The indirect, non-lexical pathway consists in grapheme-to-phoneme mappings and thus allows reading of consistent words and pseudowords. At the beginning of learning to read, the non-lexical route is the only one available for children (Ehri, 2014). With reading instruction and practice, the repeated decoding of the same words leads to the development of the lexical pathway, in which whole-word orthographic representations are stored. This route enables the reader to correctly and rapidly identify familiar words, whether they are consistent or not. It is the most frequently used route in expert readers (Ehri, 2014). At the first stages of reading acquisition, reading relies heavily on grapheme-to-phoneme mapping, phonological awareness (PA) is thus an excellent predictor of reading skills in early grades. With reading practice, reading relies more and more on the lexical route, whose efficiency is based on rapid access to phonological information from orthographic shapes. This cognitive process is thought to be highly similar to the processing stages taking place during a RAN task. Thus, RAN appeared to be a better predictor of reading outcomes in older children (Parrila et al., 2004). The dual-route approach has recently provided a comprehensive account of the factors that affect reading aloud (e.g., frequency, length, consistency, and lexicality effects) in both skilled and reading disabled children (Perry et al., 2007, 2010). Second, there are large inter-individual differences in word identification skills. Most studies involving typically developing children report a Gaussian distribution of reading skills (Plaza and Cohen, 2005; see Kirby et al., 2010 for a review). Some authors however suggested that different groups of poor readers can be distinguished among typically developing learners on the basis of their level of performance in reading (i.e. −2 vs. −1 standard deviation from the mean) and/or of associated features (i.e., poor readers showing a single PA or RAN deficit vs. those with a deficit in both PA and RAN; Cronin, 2013; de Groot et al., 2015). In fact, considerable inter-individual differences have been reported in skills associated with reading, such as PA, phonological short-term memory (PSTM), and RAN (Mann et al., 1989; Gathercole and Baddeley, 1993; Caravolas et al., 2001; Kirby et al., 2010). Here we will focus on RAN tasks, as their relation with reading skills still raises a number of issues.

Performance at RAN tasks is a reliable predictor of both concurrent and later literacy skills in children (and in adults) (i.e., Kirby et al., 2010; Georgiou et al., 2012, 2013, 2016). The strength of the RAN-reading relationship is modulated by several factors related to the characteristics of tasks used to assess RAN and reading. For example, regarding the RAN task itself, the predictive power of serial RAN on reading fluency is stronger than that of discrete RAN (Logan et al., 2011; Georgiou et al., 2013). However, recent findings suggest that discrete RAN (i.e., with items displayed one by one on a computer screen) may be an indicator of efficient reading by sight strategy (de Jong, 2011; Protopapas et al., 2013). Furthermore, the RAN task can be composed of letters, digits, pictures, or colors. In most studies, the children's performance in RAN tests using alphanumeric items has been demonstrated to be a stronger predictor of literacy skills than performance in RAN tests using pictures and colors (i.e., Schatschneider et al., 2004; Savage et al., 2008). However, significant correlations between picture/color RAN tasks performance and literacy skills have also been reported (i.e., Pauly et al., 2011; Albuquerque, 2012; Caravolas et al., 2012), and in some studies, picture RAN appeared to be more predictive than alphanumeric RAN (i.e., Arnell et al., 2009). Regarding the reading measure, RAN has been reported to be a particularly strong predictor of reading fluency (Rakhlin et al., 2014). Moreover, the relation between RAN and reading is not only dependent on the tasks properties, but also on variables related to the characteristics of the sample tested. For instance, it has been shown that the age range of the participants influences the RAN-reading relationship. Thus, as mentioned before, RAN appears to become a more powerful predictor for reading skills after Grade 3 (Parrila et al., 2004). Note that it is not clear whether the variable of interest corresponds to the chronological age of the participants, or to their degree of expertise in literacy as both usually co-vary. Reading level and age are often confounded into grade information. Age and reading level indeed share common variance, but they do not share a one to one relationship. Previous studies (de Jong, 2011) reported that the better readers of the sample were among the younger children, however classification by reading level were highly similar to classification by age, resulting in the intensive use of grade information for comparing readers. Here we orthogonalize age and reading expertise in order to tease out the contribution of age vs. reading skills to the behavioral RAN-reading relationship and to the neurophysiological changes in the discrete letter and picture RAN.

To our knowledge there are no published studies involving children and using ERP recordings during discrete letter RAN or discrete letter naming, but a few studies involving children used ERP or MEG recordings during picture naming, a task that is close to discrete picture RAN. We are not aware of a study comparing readers varying in their expertise in a typically developing sample. The two studies using ERP or MEG recordings during picture naming compared typical and dyslexic readers. Greenham et al. (2003) and Trauzettel-Klosinski et al. (2006) both reported increased error rates and longer reaction times in dyslexic participants relative to typically developed (TD) participants, but no electrophysiological correlates of these differences were observed in the picture naming task. As ERP differences were found across groups in the reading tasks, but not in picture naming, the authors suggested that the “visual” pathway is somehow preserved in dyslexic participants, at least in the early stages of picture processing. Greenham et al. (2003) hypothesized that electrophysiological differences between dyslexic and TD participants may be observed in later ERP time-windows, beyond the 500 ms analyzed in that study, possibly closer to articulation and associated with phonological processes. Consequently, an investigation of the electrophysiological correlates in a discrete RAN task should take into account longer time-intervals than those used in these studies. Regarding the effect of age on the electrophysiological correlates of picture naming, a longitudinal study (Ojima et al., 2011) using the picture-word interference task, found similar ERP components in 7 and 9 year-old children and in adults, but with shifts of latencies. The authors concluded that the differences in reaction times observed between children and adults rely on an acceleration of the processes subtending the task. Laganaro et al. (2015) compared the ERPs of typically developing 7–8 year-olds, 10–12 year-olds, and adults on an overt picture naming task. The results on the two groups of children, showed that the speeding up observed in word production does not seem to rely on a linear rescaling of all ERP components, but on a selective shortening in the time-window usually associated with lexico-phonological encoding processes.

Hence, the previous ERP results on discrete picture RAN like tasks (picture naming tasks) reported electrophysiological differences between younger and older school-age children, whereas surprisingly no modulation of ERPs was reported in picture naming tasks between dyslexic and TD readers, suggesting that reading skills do not modulate the electrophysiological correlates at least for discrete picture RAN. However, the contrast of dyslexic and typically developing children on reading level is a special case, which may not capture the RAN-reading relationship underlying typical reading acquisition. Here we take advantage of the variability within typically developing children to test with an orthogonal design in children aged 7–10 years:

1. to which extend the RAN-reading relationship is modulated (a) by the participants' age, and (b) by the participants' reading level? and
2. whether the ERP signal from discrete picture and letter RAN tasks differentiates younger and older, or poorer and better readers among typically developing children.

Contrary to behavioral approaches which do not give insight on the specific processing stages at work during a discrete RAN task and responsible for the relationship between RAN and reading, the ERP recordings during discrete RAN tasks will inform on whether age and reading level effects are sustained by different mental processes. Indeed, previous studies analyzing reaction times and error rates did not get to differentiate age and reading level (Catts et al., 2002; Parrila et al., 2004; de Jong, 2011) as the RAN-reading relationship remained constant in both cases. At the electrophysiological level, specific hypotheses can be made: age should be reflected in the ERP signal by the global acceleration of processing (Ojima et al., 2011), whereas the relationship between RAN and reading level should be observed in specific time-windows reflecting specific processing stages (i.e., lexical access and/or phonological encoding).

## Materials and methods

### Participants

Thirty two French-speaking children were selected from a larger group of 62 participants according to their age and reading skills. They were typically developing children, attending schools of the Geneva area. Recruitment was done through announcements on the University website. Children were tested individually in our lab, with two experimenters for the EEG session and one for the behavioral session. The local research ethical committee approved the study protocol. Written informed consents were collected from the children and their parents. At the end of the experimental session, the children received a small present and a voucher for their participation. The study protocol was in accordance with the Declaration of Helsinki.

Among the 32 participants, two orthogonal groups were constituted, based on age and on reading skills. There were no outliers in the selected sub-sample of children. Among participants, 8 were age-low; reading-low—8 were age-high; reading-low—8 were age-low; reading-high—and 8 were age-high; reading-high. The same 32 participants were split into two groups according to age but matched on reading skills and according to reading skills but matched on age. Poor and good readers were identified based on Text reading scores (word correctly read per minute). Poor readers obtained Text reading scores from 52 to 99 words correctly read per minutes whereas good readers obtained scores ranging from 116 to 172 words correctly read per minute. For age, two groups of 16 participants matched on reading skills, but differing on age were constituted (see Table 1). In each age group, half of the participants were good readers, and the other half were poor readers. This allowed constituting two reading skill groups (good and poor readers) of 16 participants each, who differed on reading skills for all the reading measures but were matched on age (see Table 2).

**Table 1:** Participants divided into age groups (i.e., younger and older children).

	**Age**	**Text reading (nb of words read/ minute)**	**Text reading z-score**	**Discrete reading RTs (ms)**	**Discrete reading accuracy (%)**
Young children	8.0 (±0.69)	104.88 (±39.92)	0.8 (±0.4)	835 (±127)	82 (±10)
Older children	9.68 (±0.48)	119.69 (±40.41)	0.88 (±0.42)	810 (±120)	88 (±9)
*P*-value	< 0.001	>0.31	>0.60	>0.58	>0.10

**Table 2:** Participants divided into reading skills groups (i.e., poor and good readers).

	**Age**	**Text reading (nb of words read/ minute)**	**Text reading z-score**	**Discrete reading RTs (ms)**	**Discrete reading accuracy (%)**
Poor readers	8.66 (± 1.17)	76.5 (± 16.26)	0.5 (± 0.18)	878 (± 104)	79 (± 10)
Good readers	9.02 (± 0.88)	148.06 (± 18.67)	1.18 (± 0.24)	768 (± 117)	91 (± 5)
*P*-value	>0.34	< 0.001	< 0.001	< 0.001	< 0.001

### Task and material

#### Reading measure

##### Text reading

Text reading was assessed by using the test “Monsieur Petit” extracted from the “Evaluation de la Fluence en Lecture” battery (Lequette et al., 2008). In this test, children are instructed to read aloud as fast and accurately as possible a text containing 24 lines and 352 words. The experimenter asks them to stop after 1 min. The text reading score is the number of words correctly read within 1 min.

##### Discrete reading

Sixteen monosyllabic words were selected from the French lexical database Manulex (Lété et al., 2004). All words were four to six letter long with an average print lexical frequency of 115.6 per million. Changing at least two letters in the set of words created eight orthographically legal and pronounceable pseudowords. The stimuli were displayed on a computer screen using the software E-prime (E-studio). Each trial began with a fixation cross presented for 500 ms at the center of the screen. The fixation cross was then replaced by a gray screen for 200 ms, followed by the word for 2000 ms in the middle of the screen. The fixation cross-picture sequence was manually triggered by an experimenter sitting behind the child. The children were asked to read aloud the words and pseudowords as fast and accurately as possible. The task was divided into two parts: word reading (with the 16 words repeated each 5 times) and pseudoword reading (with the eight pseudowords repeated each five times). By dividing the number of correct responses by the mean reaction time a composite discrete reading score was computed.

### Phonological awareness

The phonological awareness tasks were borrowed from the Odedys battery (i.e., spoonerism task; Jacquier-Roux et al., 2002), and from the Isadyle battery (i.e., initial phoneme deletion task; Piérart et al., 2005). The two PA scores correspond to the number of correct responses in each task (out of 8 trials for the spoonerism task, and 10 trials for the phoneme deletion task).

### RAN

#### Serial tasks: Picture and Letter

For both tasks, the child was asked to name as fast and accurately as possible the items displayed on an A4 sheet (landscape orientation). Responses were digitally recorded. A speech analysis software (Praat: doing phonetics by computer, Boersma and Weenink, 2013) was used to measure the total time taken by the child to name all the items for each grid.

#### Pictures

Sixteen black and white drawings and their corresponding modal names were selected from French databases (Alario and Ferrand, 1999; Bonin et al., 2003). The stimuli corresponded to 16 words with an age of acquisition range of 1.31–2.95 on a five-point scale (1: learned between 0 and 3 years; 4: learned between 9 and 12 years) and high name agreement (mean = 93.6 %) to ensure that the children give the same name for a same picture. They were displayed on two A4 sheets, with three repetitions of each item (24 stimuli per grid). A familiarization trial with all pictures and their corresponding modal names was carried out prior to running the experiment.

#### Letters

Sixteen letters were selected as a function of to their syllable frequency and letter frequency characteristics. Stimuli were displayed on two A4 sheets, with three repetitions of each item (24 stimuli per sheet).

#### Discrete tasks

The same stimuli as those used in the serial RAN tasks were displayed one by one on a computer screen using the software E-prime (E-studio). These tasks were performed under EEG recording. Each trial began with a fixation cross, presented for 500 ms in the center of the screen, then a gray screen for 200 ms followed by the stimulus. The duration of the presentation varied across tasks (i.e., 2000 ms for the pictures, and 800 ms for the letters). In order to avoid recording EEG when the signal was noisy due to the child's movements, an experimenter sitting behind the child, who was in visual contact with the other experimenter monitoring the online EEG signal, manually triggered the trials. The children were asked to name aloud the pictures and letters as fast and accurately as possible. Word productions were digitally recorded and production latencies (i.e., the time separating the onset of the picture and the onset of the speech wave) were systematically computed with a speech analysis software (Check-Vocal, Protopapas, 2007). The discrete RAN scores comprise the average RTs and the number of correct responses per stimuli type.

### EEG acquisition and pre-analyses

EEG was recorded continuously during discrete RAN tasks using the Active-Two Biosemi EEG system (Biosemi V.O.F. Amsterdam, Netherlands) with 64 channels covering the entire scalp. Signals were sampled at 512 Hz (filters: DC to 104 Hz, 3 dB/octave slope). The common mode sense (CMS; active electrode)—driven right leg (CMS-DRL) is the online reference in the Biosemi system. Offline, ERPs were then bandpass-filtered to 0.2–30 Hz and notch-filtered to 50 Hz and re-referenced to the average reference. Epochs were extracted locked to the stimulus (the word, the picture, the letter) with different duration according to the production latencies in each task. Average reaction times were 955 ms for picture naming and 683 ms for letter naming. Epochs were extracted from −50 to 400 time-frames (i.e., 798 ms) in the discrete picture RAN and epochs from −50 to 250 time-frames (i.e., 488 ms) for the discrete letter RAN. Epochs contaminated by eye blinking, eye-movements, movements or other noise were rejected and excluded from averaging after visual inspection. Baseline correction was applied based on the 100 ms pre-stimulus interval. Only trials with correct responses and valid RTs were retained. Epoch extraction and averaging was computed for each participant using the Cartool software (Brunet et al., 2011). As a result, an average of 64 averaged trials per participant and per task entered the ERP analyses (range: 42–78). Electrodes with signal artifacts were interpolated using 3-D splines interpolation (Perrin et al., 1987), with an average of eight sites interpolated for each participant.

## Results

### Behavioral results

In order to diminish the number of variables, the *z*-score values of the six RAN indexes (serial picture RAN total time, serial letter RAN total time, discrete picture RAN mean reaction time and number of correct responses, discrete letter RAN mean reaction time and number of correct responses) were entered into a Factorial analysis (principal component using promax rotation with Kaiser normalization, SPSS software)^1^. Two components were extracted representing a total of 66.9% of explained variance. As can be seen in Table 3, the loadings of the first component, which explains 41.4% of variance, are mostly related to the picture RAN variables. This component was therefore labeled Picture RAN factor. The second component, explaining 25.4% of variance, is more strongly related to the letter RAN variables, and was consequently labeled Letter RAN factor. A similar analysis was performed with the two Phonological Awareness tasks, the Phoneme deletion task and the Acronym task (*z*-score values). The Factor extracted explains 64.4% of the variance, and the loading of each variable was 0.802.

**Table 3:** Structure matrix for the principal component analysis performed on the RAN variables.

	**Component**	
	**1**	**2**
Serial RAN Picture total time	0.876	0.198
Serial RAN Letter total time	0.308	0.870
Discrete RAN Picture Correct responses	−0.838	−0.203
Discrete RAN Picture Mean RT	0.607	0.066
Discrete RAN Letter Correct responses	−0.542	−0.662
Discrete RAN Letter Mean RT	−0.082	0.848

We then tested whether these factors would allow discriminating the participants as a function of their age and reading level. We thus performed a multiple analysis of variance comparing the performance of the participants by Age (younger vs. older) and Reading level (good vs. poor) with the three factors representing the RAN and the PA tasks as dependent variables. The results reveal a significant main effect of Age, *F*_(3, 26)_ = 4.207, *p* = 0.015, and of Reading level, *F*_(3, 26)_ = 4.513, *p* = 0.011. The Age X Reading level interaction does not reach significance (*p* > 0.3). Table 4 reports the effects of Age and Reading level variable by variable. It can be seen that the effect of Age is significant only on the Letter RAN factor. By contrast, the effect of Reading level is highly significant on the Picture RAN factor, and a trend is observed on the other two factors.

**Table 4:** Results of the multiple analysis of variance as a function of Age and Reading level per variable.

**Factor**	**Variable**	***F***	***df***	***p***	***etasqu***
**AGE**					
	Factor RAN Picture	0.129	1.28	0.722	0.005
	Factor RAN Letter	12.560	1.28	0.001	0.310
	Factor PA	0.190	1.28	0.666	0.007
**READING LEVEL**					
	Factor RAN Picture	10.822	1.28	0.003	0.279
	Factor RAN Letter	3.177	1.28	0.086	0.102
	Factor PA	3.373	1.28	0.077	0.108

*The Age by Reading level interaction does not reach significance on any of the variables, all ps > 0.13*.

Finally, two regression analyses were performed to test which variables predicted reading level. In both analyses, the predictors were the two RAN factors, the PA factor and Chronological age. In the first analysis, the dependent variable was Text reading fluency. The results show that this variable is only predicted by the Letter RAN factor, *F*_(1, 30)_ = 6.64, *p* = 0.015, *R*^2^ = 0.194. The second analysis had a composite measure of discrete reading (which combines RT and number of correct responses) as a dependent variable. The results indicate that both the Picture RAN factor, *F*_(1, 30)_ = 10.90, *p* = 0.002, *R*^2^ = 0.242, and Chronological Age, *F*^Δ^_(1, 29)_ = 7.17, *p* = 0.012, *R*^2Δ^ = 0.145, contribute to explain the variance of discrete reading.

### ERP results

The ERPs of Discrete picture RAN and Discrete letter RAN were subjected to standard waveform analysis to determine the time periods of amplitude differences between age groups and reading-performance-groups. This analysis was performed on all electrodes and data-points. One-way analyses of variance (ANOVA) were computed on amplitudes of the evoked potentials between groups using the STEN toolbox (developed by Jean-François Knebel; http://www.unil.ch/line/home/menuinst/about-the-line/software–analysis-tools.html). Only differences over at least four clustered electrodes and extending over at least 10 consecutive time-frames (i.e., 20 ms) were retained with an alpha criterion of 0.05.

**Figure 1:**
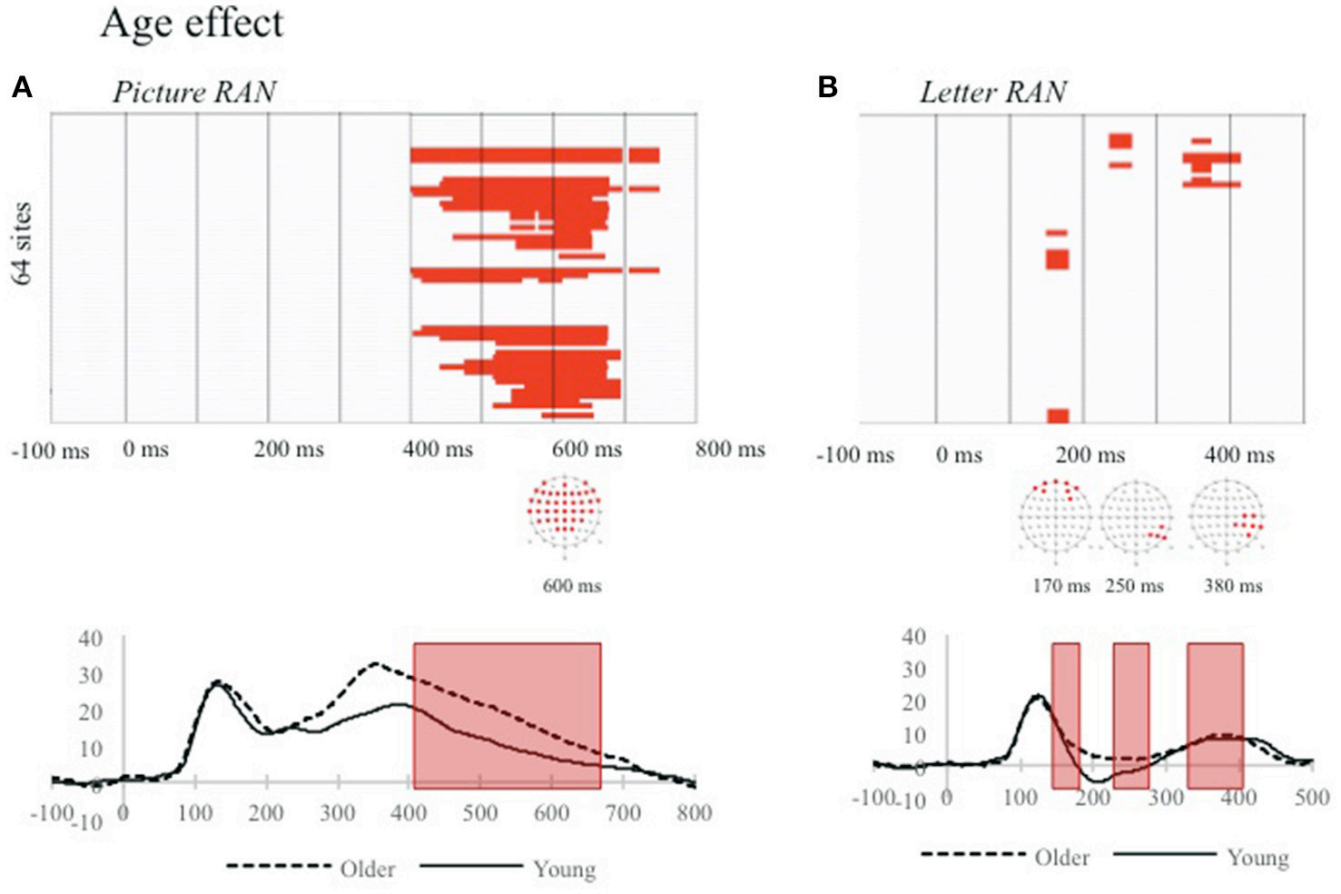
Significant differences on ERP waveform amplitudes for each electrode (y axes) and time-point (x-axes) between younger and older children for the two discrete RAN tasks: discrete picture RAN **(A)** and discrete letter RAN, **(B)**. Only differences over at least four clustered electrodes and 10 time frames, with an alpha criterion of 0.05 are displayed in red. The channel yielding the significant differences of amplitudes and an example waveform is displayed under each graph (O1) with time-windows of significant effects displayed with a red shape. (For interpretation of the reference to color in this figure legend, the reader is referred to the web version of this article).

Figure 1 shows time points of significant amplitude differences between younger and older children for the two RAN tasks. For Discrete picture RAN (Figure 1A), significant differences appeared between younger and older children from 400 ms after stimulus presentation, and extend until 750 ms. Concerning Discrete letter RAN (Figure 1B), significant differences across age-groups are observed from 160 to 190 ms and from 350 to 410 ms after stimulus presentation. In both tasks amplitudes were more negative on posterior electrodes (see O1 displayed on Figures 1A,B) for the younger group.

**Figure 2:**
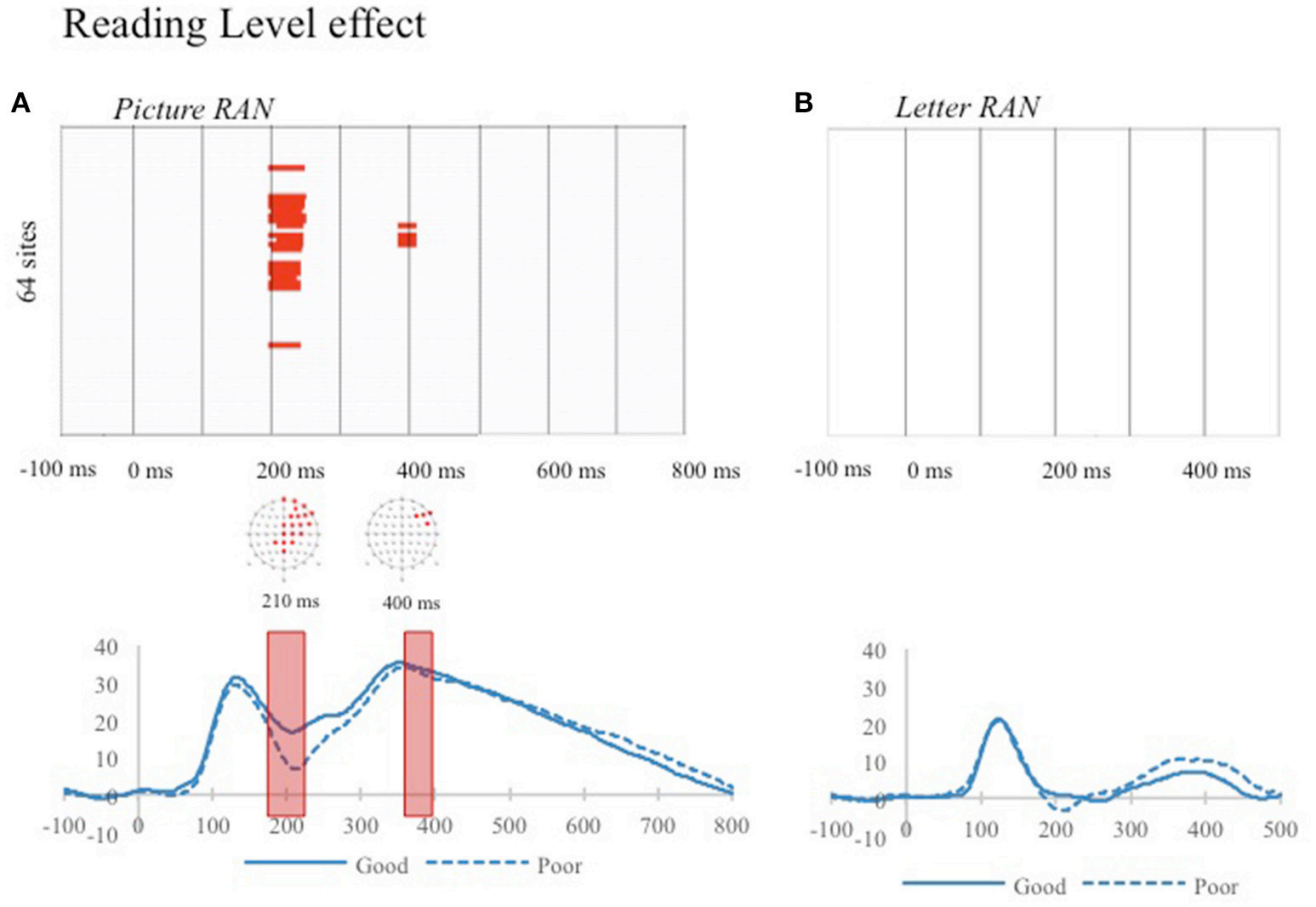
Significant differences on ERP waveform amplitudes s for each electrode (y axes) and time-point (x-axes) between poor and good readers for the two discrete RAN tasks: discrete picture RAN **(A)** and discrete letter RAN **(B)**. Only differences over at least four clustered electrodes and 10 time frames, with an alpha criterion of 0.05 are displayed in red. The channel yielding the significant differences of amplitudes and an example waveform is displayed under the graph (O1) with time-windows of significant effects displayed with a red shape. (For interpretation of the reference to color in this figure legend, the reader is referred to the web version of this article).

Figure 2 shows the time-points of significant amplitude differences between good and poor readers. In the discrete picture RAN task (Figure 2A), significant differences between good and poor readers appeared in the N2 time-interval (i.e., 200–250 ms) on a large cluster of central-anterior channels and in a short later time-window (i.e., from 380 to 410 ms after stimulus presentation) on a small cluster of electrodes In the N2 time-interval amplitudes were more negative on posterior electrodes for poor readers (see Figure 2A). No significant differences between good and poor readers were found in the Discrete letter RAN task (Figure 2B).

## Discussion

In this study we investigated whether the RAN-reading relationship is modulated (a) by the participants' reading level, and (b) by the participants' age among a sample of typically developing children, and whether the ERP signal from a discrete RAN task differentiates younger and older, and/or poor and good readers. For this purpose, we developed a design in which two groups of children were matched on age to investigate the impact of reading skills, or on reading skills to investigate the impact of age.

### Age effect

The behavioral results revealed that young and older children differ in their performance on the letter RAN, with slower naming times for younger children, whereas no age differences appeared on the picture RAN and the PA factors on groups matched on reading skills. The effect of age limited to the letter RAN task advocates for a stimulus effect between younger and older children. Given that both Age groups do not differ in reading level, this effect is more likely dependent on the duration of exposure to the written code than on reading expertise *per se*. In any case, these results suggest that the letter RAN is more sensitive to age differences than the picture RAN. Interestingly, our results show that PA skills do not seem to vary according to the age of the participants, at least within the age range tested in this study. Actually, participants perform very well in PA tasks, resulting in high scores and low variability within the sample, which can explain the absence of PA effect according to age. It could be the case that PA accuracy cannot differentiate groups of participants in a typically developed sample. Indeed, children perform too well in PA tasks after the early grades. The difference in PA skills between groups could be expressed at the reaction time level, as every children can give the right answer, but older ones are faster.

We found also specific time-intervals in the ERP signal in Discrete RAN tasks modulated by age. In the discrete letter RAN task, the first differences between younger and older children appeared in the N170 time-window with larger amplitudes for younger children. This result is in line with a stronger sensitivity to print in older children (Maurer et al., 2006) and with the behavioral results reported earlier. Crucially, we found more extended and later (from 400 to 750 ms) electrophysiological differences between younger and older children in the discrete picture RAN. Overall, these results suggest that the entire time-course of discrete picture and letter RAN develops across age.

### Reading skill effect

At the behavioral level, good and poor readers differed mainly in their performance on the picture RAN factor, although a trend was also observed on the letter RAN and on the PA factors. These results suggest that picture RAN is a better index of reading level variance than alphanumeric RAN, a result that is in line with those of Arnell et al. (2009). It however runs against the dominant view that alphanumeric RAN is a stronger predictor of reading skills than RAN tasks using other stimuli (Manis and Doi, 1995; Misra et al., 2004; Schatschneider et al., 2004; Savage et al., 2008). Direct comparison of the present results with these previous studies should nevertheless be done with caution given the fact that our picture RAN factor is a composite measure that involves mostly, but not exclusively, picture RAN, and is based both on serial and discrete versions of the RAN task. In the two regressions analysis, we investigated which factor predicts reading skills both in terms of text reading and of discrete reading. Results showed a clear-cut difference between the two types of reading assessments. Indeed, text reading variance is predicted by the Letter RAN factor only whereas discrete reading variance is predicted by both the Picture RAN factor and age.

The previously reported alphanumeric superiority at the behavioral level in the RAN-reading relationship could be explain by the type of reading tasks used in previous studies. Indeed, previous studies mostly used text reading to address reading fluency, which may led to the systematic distinction between predictive powers of letter and picture RAN (see Kirby et al., 2010 for review). During text reading, participants rely on context to predict the next words, the prediction of the words to come is based on both context and first letter of the word. Thus, it is expected that this type of processes relate more with letter RAN. During a discrete reading task, the use of context and first letters to guess what word will be displayed next is impossible. Therefore, discrete reading task are by nature more similar to picture naming task as they both require the retrieval of a phonological form from visual information taken at once. The present results advocate for caution when selecting the reading task according to the hypothesis to be tested, as text and discrete reading appear to be different tasks by nature.

Again, the PA factor does not predict reading skills variance in our sample. Cronin (2013) argued that the long lasting predictive power of PA across elementary grades is specific to English, which behaves as an “outlier” among European languages (Share, 2008). Studies in transparent orthographies (Wagner et al., 1994; Manis et al., 1999; Wimmer et al., 2000; Lepola et al., 2005; Verhagen et al., 2008) reported that PA predicts reading skills only through second grade, which is similar to our results. Note that French is considered as a mid-opaque orthographic system.

Our results suggest that letter and picture RAN do not address the exact same processing stages as they relate differently to reading tasks. Moreover, it confirms that the format of the reading task seems to be crucial when investigating the RAN-reading relationship as advocated by de Jong (2011). Indeed, previous studies reporting an alphanumeric superiority in the RAN-reading relationship (Schatschneider et al., 2004; Savage et al., 2008) are mostly based on text reading or on a reading assessment combining both text and word reading.

When children are divided into groups according to their reading level but matched on age, group differences in ERPs are limited to the discrete picture RAN task. In the discrete picture RAN ERPs, poor readers exhibited larger amplitudes than good readers around 200 ms, corresponding to a N170/N200 component and lower amplitudes around 400ms after the picture onset on screen. The N170 interval in picture naming has been associated with recognition of the picture and conceptual/semantic processes (Schendan and Kutas, 2003; Indefrey, 2011). The second time-window falls within a P2 component (see Figure 2), although it is clearly delayed in the present study relative to studies with adult participants. A similar delay of component was previously reported in studies with children (Trauzettel-Klosinski et al., 2006; Laganaro et al., 2015). If one proportionally rescales adult's time-course estimates taking this delay into account, the second positive component peaking in the youngest children around 400 ms could be interpreted as a P2. Modulations of amplitudes within the P2 time-interval have been previously associated with frequency effects in picture naming studies involving adults (Strijkers et al., 2011) and the P2 component has been associated with lexical selection (Indefrey, 2011). The differences in waveform amplitudes around the P2 component and beyond may therefore reflect differences in lexical selection and phonological encoding between good and poor readers.

The present results diverge from those of previous studies using ERP/MEG recordings during picture naming with groups of children varying in their reading expertise (Greenham et al., 2003; Trauzettel-Klosinski et al., 2006) which did not report ERP differences between groups (see Introduction). Here we found specific time-intervals in the picture naming task differentiating poor and good readers. Contrary to the hypothesis made by Greenham and colleagues that ERP differences between time-windows differentiating TD and dyslexic participants should appear beyond 500 ms after stimulus presentation, we reported differences as soon as the N2 component. It should be noted that comparison between the present results and results reported by Greenham et al. (2003) should be done with caution. In fact, Greenham and colleagues used a picture-word interference paradigm, which is different from the bare picture naming task used here. Also, previous studies had rather small samples sizes (i.e., 8–13 subjects in each group), which can explain the lack of differences between groups in picture naming.

### Age and reading skills in the RAN-reading relationship

Previous studies reported an alphanumeric superiority effect on the RAN-reading relationship (Manis and Doi, 1995; Misra et al., 2004; Schatschneider et al., 2004; Savage et al., 2008). Our behavioral and ERP results converge in suggesting that the alphanumeric superiority is a matter of age more than a matter of reading efficiency, and is probably subtended by a longer exposure to printed information. The age by reading level interaction did not reach significance for any of the three factors entered in the analysis, which indicates that reading level and age effects are fairly independent from each other. In addition only age modulated ERPs in the letter RAN. By contrast, picture RAN performance and specific processing stages indexed by the ERP signal in picture RAN are highly related to both reading skills and age. Taken together the present results at both the behavioral and the electrophysiological levels give new insights on the RAN-reading relationship. First, it clearly appear that age and reading efficiency, even though they co-vary, do not represent the same concept. Apparently, age cannot be used as a proxy for reading efficiency, at least in French. Secondly, the alphanumeric superiority previously reported in the literature on the RAN-reading relationship seems to be balanced by the present findings suggesting that alphanumeric RAN captures cognitive changes related to age but not to reading level. Indeed, alphanumeric RAN scores reflect the degree of automation of closed-class stimuli (i.e., letters). Moreover, knowing the letter names is not a good indicator of reading skills once formal reading instruction began, but knowing the letter-phoneme correspondence is (Blaiklock, 2004). Third, we propose that picture RAN is related to reading level because of lexical access and lexico-phonological binding stages. Poor and good readers differ specifically on two components: the N170 and the P2, reflecting early lexical access and lexico-phonolgical binding in the reading literature (Maurer et al., 2006). In the picture naming time-course, the P2 component is usually associated with lexical access (Indefrey, 2011) and the N170-like component seems to be specific to children (Laganaro et al., 2015). Here we report differences between good and poor readers in these two specific time-intervals, suggesting that the processing stages taking place between 200 and 500 ms in picture RAN are the cornerstone of the RAN-reading relationship. Moreover, we argue that lexical access stage in not present in letter naming—at least not in the same sense as in picture naming or reading—which explains the absence of reading effect on letter RAN (Grainger et al., 2008; Madec et al., 2012).

## Conclusion

To our knowledge, this study is the first to compare younger and older children as well as good and poor readers in a sample of typically developing children on their performance in various RAN and reading tasks and to report ERP modulation by age and reading level on discrete RAN tasks. Discrete letter RAN processes appeared to be modulated by the participant's age, whereas processes tackled by the picture RAN task seem to be modulated both by the participant's reading expertise and by age. This suggests that there are specific processes tackled by the discrete picture RAN task that are likely to constitute the cornerstone of the RAN-reading relationship whereas discrete letter RAN tasks are sensitive to the duration of exposure to the written code. Future studies dedicated to the investigation of the RAN-reading relationship should investigate which cognitive processes underlie these specific relationships between RAN task format and age vs. reading skills.

## Ethics statement

This study was carried out in accordance with the recommendations of the University of Geneva Ethic Committee with written informed consent from all subjects. All subjects gave written informed consent in accordance with the Declaration of Helsinki. The protocol was approved by the Faculty of Psychology research ethical committee.

## Author contributions

Each author has participated sufficiently in the work to take responsibility for certain portions of the manuscript's content. MC: Made substantial contributions to conception and design, data collection and analysis and interpretation of the data. GM: Made significant contributions to data collection and analysis and to interpretation of the data; ML: Made considerable contribution to conception and design and to analysis and interpretation of the data. PZ: Made significant contribution to conception and design and to analysis and interpretation of the data.

### Conflict of interest statement

The authors declare that the research was conducted in the absence of any commercial or financial relationships that could be construed as a potential conflict of interest.

## Footnotes

^1^Serial and Discrete RAN were significantly inter-related. They were entered together into the factorial analysis.
